# Ploidy dynamics increase the risk of liver cancer initiation

**DOI:** 10.1038/s41467-021-21897-8

**Published:** 2021-03-25

**Authors:** Miryam Müller, Stephanie May, Thomas G. Bird

**Affiliations:** 1 Cancer Research UK Beatson Institute, Glasgow, UK; 2 MRC Centre for Inflammation Research, The Queen’s Medical Research Institute, University of Edinburgh, Edinburgh, UK

**Keywords:** Cancer genomics, Chromosome segregation

## Abstract

Liver cancer typically arises after years of inflammatory insults to hepatocytes. These cells can change their ploidy state during health and disease. Whilst polyploidy may offer some protection, new research shows it may also promote the formation of liver tumours.

In the adult human, most cells are diploid, containing a single pair of each chromosome (2*n*). However, some specialised cells possess multiple complete pairs of chromosomes; a state called polyploidy. Hepatocytes, the primary functional cell of the liver, exist in diploid and polyploid states^1^. Hepatocytes increase ploidy through acytokinetic or cytokinetic mitoses, instead of relying on cell fusion. After birth, progressively polyploid (first 4*n*, then ≥6*n*) hepatocytes are formed. A combination of polyploid hepatocytes, varying in both nuclear and cellular polyploidy, form a major fraction of the healthy adult liver; ~30% in man and >90% in mice^1^. Ploidy in hepatocytes is balanced by the “ploidy conveyor” in which increasingly polyploid cells are more likely to reduce their ploidy towards diploidy. Ploidy reduction occurs through mitosis either bipolar or multipolar, leading to the generation of two or more daughter cells^2^. Despite evidence that, in general, polyploidisation can be protective against cancer, the question of whether it also has a darker side has remained, particularly in the formation of hepatocellular cancer (HCC).

The liver has an exceptional regenerative capacity reflective of its exposure to repeated hepatocellular damage due to its anatomical site and functional role in detoxification. The liver may have evolved polyploidisation to both protect from frequent genotoxic insults and to allow rapid regenerative flexibility. All hepatocytes can regenerate, but one rapid mechanism is through ploidy reduction. Cellular ploidy decreases after liver resection (partial hepatectomy) and ensuing regeneration through polyploid hepatocytes entering the M phase without prior S phase^3^. Here, the reduction of multinuclear hepatocytes is balanced by the formation of mononuclear polyploid cells^4^. However, chronic injury is associated with increased polyploidy. While polyploidy is associated with cellular dysfunction and genetic instability in some cells, it may also buffer for genetic^4–7^ and transcriptional^8^ instability. Buffering of genetic instability occurs through the possession of multiple copies of chromosomes preventing the dominant effect of mutation at a single genetic locus (Fig. 1). This genomic redundancy particularly protects from loss of heterozygosity (LOH) of tumour suppressor genes.

**Fig. 1:**
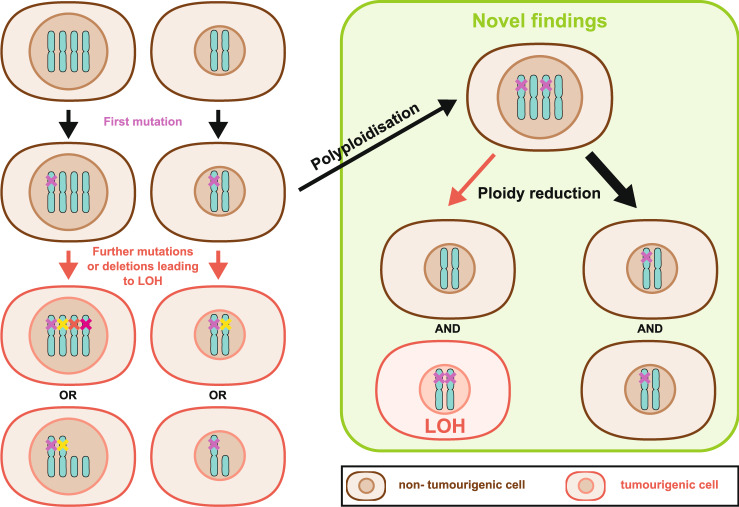
Ploidy and loss of heterozygosity. Polyploid hepatocytes are believed to be protected from loss of heterozygosity (LOH) through the possession of multiple copies of each chromosome. The probability of independent mutations or deletions occurring across multiple copies of each chromosome is very rare compared to diploid cells. Loss of heterozygosity refers to the complete loss of function of a single tumour suppressor gene in all chromosomes either by mutation or deletion after prior mutation at a single locus. Complete loss of all functional copies of a tumour suppressor gene may impose dysregulated proliferation and tumourigenic potential. Matsumoto et al.^11^ and Lin et al.^12^ suggest that particularly for liver cancer dynamic changes in ploidy, first polyploidisation then ploidy reduction of cells containing heterozygote loss of a tumour suppressor gene is associated with tumour initiation. Hypothetically mis-segregation of mutant tumour suppressor genes may occur during the process of ploidy reduction leading to LOH. Typically, while chromosomes are segregated faithfully (thick black arrow), which does not escalate the tumour risk, due to the proliferative advantage that LOH may impose even rare mis-segregation events can have a dramatic consequence and could lead to carcinogenesis.

HCC is typically a complication of chronic liver disease. Progressive genetic damage to hepatocytes leads to activation of genetic drivers (oncogenes) or the loss of tumour suppressor genes causing cancer formation. HCC may be either diploid or polyploid, the latter typically associated with the loss of the p53 tumour suppressor in late-stage disease. In other cancers, polyploidy is associated with tumour formation. However, little is known about the role that ploidy dynamics play in HCC initiation. Ploidy reduction is rarely associated with aneuploidy^9^, and generally, ploidy reduction in hepatocytes results in faithful chromosome segregation^10^. In mouse models, when ploidy is manipulated, increasingly polyploid livers develop fewer HCC and diploid hepatocytes are more at risk of LOH^4–7^. However, recently Matsumoto et al.^11^ and Lin et al.^12^ demonstrate the importance of ploidy dynamics in the formation of HCC in mice without directly manipulating hepatocyte ploidy.

Lin et al. used a diethylnitrosamine (DEN)-induced carcinogenic model to investigate the role of polyploidisation during the transformation of hepatocytes into preneoplastic HCC lesions^12^. DEN is a widely used hepatotoxin and carcinogen, inducing HCC in rodents, albeit with debatable relevance to human HCC^13^. DEN was administered perinatally causing a zonal liver injury and mutagenic DNA adducts. The authors then observed increased hepatocyte polyploidisation in and around areas damaged by the carcinogen. Importantly, hepatocytes from the injured zone formed spheres in culture and preferentially formed tumours in a transplant assay unlike those from other areas. Foci, reminiscent of preneoplastic foci in humans, formed within the damaged liver. These foci contained small, presumably diploid cells, unlike their surrounding polyploid non-neoplastic tissue. Linking these foci to originating polyploidy hepatocytes and resultant tumours was not possible, but these data suggest that ploidy increase followed by reduction is associated with preneoplastic foci establishment. However, the authors were able to associate a link from early to late-stage disease mechanistically. They found an Aurkb-driven abscission (cell separation) failure promoted polyploid hepatocyte formation. Inhibiting Aurkb resulted in reduced early polyploidy formation and also reduced the formation of both preneoplastic foci and late-stage tumours; therefore, demonstrating that polyploidisation and its subsequent reduction promotes carcinogenesis in this model. Interestingly, Aurkb was also overexpressed in human fatty liver disease, progressive liver fibrosis and late-stage HCC.

An elegant study by Matsumoto et al. examined ploidy reduction in early tumourigenesis^11^. Here, the authors used their novel multicolour lineage tracing system^10^ to trace the tumourigenic potential of polyploid hepatocytes alongside the ploidy status of their progeny. In this model, upon induction of Cre-recombinase, a reporter, which is only expressed from one chromosome in diploid cells, expresses one of the multiple fluorescent reporters. However, as polyploid hepatocytes possess multiple chromosome copies, multiple different fluorescent reporters may be co-expressed^10^. This enables detection, capture and tracing of polyploid hepatocytes and their progeny over time. Exploiting this technique, the authors examine the regenerative and tumourigenic potential of polyploid hepatocytes in a fumarylacetoacetate hydrolase transplant model^14^. In this model, where endogenous metabolically deficient hepatocytes are outcompeted by fitter transplanted cells, they first showed that polyploid hepatocytes frequently undergo ploidy reduction during liver repopulation. They then show that upon repeated proliferation cycles, through serial transplantation, this ability of polyploid hepatocytes to undergo ploidy reduction was lost; a process associated with loss of multi-nucleation. The mechanism by which this adaptation occurs remains elusive. In tumourigenic assays, using either introduction of oncogenes or a variety of toxins inducing HCC, they show that bicoloured polyploid hepatocytes often undergo ploidy reduction during early carcinogenesis. Moreover, when tumour suppressor loss was assessed in competitive tumourigenic assays, the overwhelming majority of initiating hepatocytes underwent ploidy reduction during cancer initiation. Overall, a striking finding was that ploidy reduction plays a fundamental role in LOH-induced cancer.

Previous studies have shown that polyploid hepatocytes have reduced tumourigenic potential. These two new studies show that the situation is more complex and polyploid hepatocytes are not protected from oncogenesis, but can promote tumour initiation through ploidy reduction. This leads to a fundamental question of whether some ploidy is “good” (physiological polyploidy) and helps with regeneration, whereas other ploidy is “bad” (pathological polyploidy) and has an intrinsic malignant potential? The suggestion from Matsumoto et al. that cells can adapt to repeated proliferation and prevent ploidy reduction would suggest that this is indeed the case. Previous studies using genetic perturbation to induce either increased or reduced ploidy have linked increased polyploidy to reduced tumour risk^4–7^. These new studies now suggest a mechanism through which a change in ploidy, particularly ploidy reduction, is associated with carcinogenesis.

Intriguingly, Matsumoto et al. show that ploidy reduction plays a stronger role in tumour suppressor gene loss-driven tumourigenesis than oncogene-induced carcinogenesis. The redundant genome of polyploid cells is thought to safeguard against LOH, protecting from malignant transformation. However, it appears that the act of ploidy reduction may invert this protective effect. If faithful chromosome segregation were to fail, even rarely, then ploidy reduction could easily lead to homozygous loss of tumour suppressors and promote preneoplastic foci formation (Fig. 1). Indeed, Matsumoto et al. observed unfaithful chromosome segregation in an experiment in which LOH occurred for an enzyme required for selective disadvantage, possibly through aneuploidy, but potentially through miss-segregation, consistent with previous studies^15,16^. If this is the case, in light of the findings from these two new studies, then the combination of a proliferative burst at a time of genetic instability, both mutational and through multipolar mitosis, would be particularly dangerous.

Ageing also plays a fundamental role in polyploidisation. New-born rodent hepatocytes are diploid and polyploidisation starts after weaning. Aurkb plays a role in failed cytokinesis typically observed during early developmental polyploidisation^17^. In the Lin et al. study, DEN was given preweaning while hepatocytes begin polyploidisation. However, is Aurkb and a subsequent abscission failure equally relevant for the development of HCC in later life where polyploidisation is mostly driven by endoreplication? Evidence linking Aurkb overexpression in HCC to advanced tumour stage and high-grade histology^18^ not only advocates a relevance of failed cytokinesis in adult tumourigenesis but it also suggests that tumours co-opting mechanisms seen in early developmental polyploidisation may be more aggressive as a result.

In adulthood, the proportion of polyploid cells in the liver is greater in the mouse than in human. Therefore, findings from rodent models may over-interpret the human relevance of ploidy-related tumourigenesis. Nonetheless, these are important models and the idea put forward by Matsumoto et al. that prolonged proliferation stabilises polyploid cells prevents ploidy reduction and reduces malignant transformation is intriguing. Dissecting the mechanism through which this occurs might uncover therapeutic opportunities for the prevention of HCC. Similarly, the processes discussed above could serve as biomarkers for future cancer risk. In summary, both polyploidisation and, in particular, ploidy reduction carries the potential for malignant transformation, and yet both processes have clearly been selected for maintaining liver physiology. These new studies have shed new light on this topic highlighting a potentially critical role for tumour formation in the liver by ploidy reduction.
